# Effect of the surgical approach on survival outcomes in patients undergoing radical hysterectomy for cervical cancer: A real‐world multicenter study of a large Chinese cohort from 2006 to 2017

**DOI:** 10.1002/cam4.3287

**Published:** 2020-07-06

**Authors:** Chenyan Guo, Xiaoyan Tang, Yan Meng, Ying Zhang, Xuyin Zhang, Jingjing Guo, Xiaohong Lei, Junjun Qiu, Keqin Hua

**Affiliations:** ^1^ Department of Gynecology, Obstetrics and Gynecology Hospital Fudan University Shanghai China; ^2^ Shanghai Key Laboratory of Female Reproductive Endocrine‐Related Diseases Shanghai China

**Keywords:** cervical cancer, laparotomy, matching, minimally invasive surgery, radical hysterectomy, survival outcome

## Abstract

**Objective:**

To compare survival outcomes of minimally invasive surgery (MIS) and laparotomy in early‐stage cervical cancer (CC) patients.

**Methods:**

A multicenter retrospective cohort study was conducted with International Federation of Gynecology and Obstetrics (FIGO, 2009) stage IA1 (lymphovascular invasion)‐IIA1 CC patients undergoing MIS or laparotomy at four tertiary hospitals from 2006 to 2017. Propensity score matching and weighting and multivariate Cox regression analyses were performed. Survival was compared in various matched cohorts and subgroups.

**Results:**

Three thousand two hundred and fifty‐two patients (2439 MIS and 813 laparotomy) were included after matching. (1) The 2‐ and 5‐year recurrence‐free survival (RFS) (2‐year, hazard ratio [HR], 1.81;95% confidence interval [CI], 1.09‐3.0; 5‐year, HR, 2.17; 95% CI, 1.21‐3.89) or overall survival (OS) (2‐year, HR, 1.87; 95% CI, 1.03‐3.40; 5‐year, HR, 2.57; 95% CI, 1.29‐5.10) were significantly worse for MIS in patients with stage I B1, but not the cohort overall (2‐year RFS, HR, 1.04; 95% CI, 0.76‐1.42; 2‐year OS, HR, 0.99; 95% CI, 0.70‐1.41; 5‐year RFS, HR, 1.12; 95% CI, 0.76‐1.65; 5‐year OS, HR, 1.20; 95% CI, 0.79‐1.83) or other stages (2) In a subgroup analysis, MIS exhibited poorer survival in many population subsets, even in patients with less risk factors, such as patients with squamous cell carcinoma, negative for parametrial involvement, with negative surgical margins, negative for lymph node metastasis, and deep stromal invasion < 2/3. (3) In the cohort treated with (2172, 54%) or without adjuvant treatment (1814, 46%), MIS showed worse RFS than laparotomy in patients treated without adjuvant treatment, whereas no differences in RFS and OS were observed in adjuvant‐treatment cohort. (4) Inadequate surgeon proficiency strongly correlated with poor RFS and OS in patients receiving MIS compared with laparotomy.

**Conclusions:**

MIS exhibited poorer survival outcomes than laparotomy group in many population subsets, even in low‐risk subgroups. Therefore, laparotomy should be the recommended approach for CC patients.

## Graphical abstract

MIS exhibited poorer survival outcomes than laparotomy in many population subsets, even in low‐risk subgroups. Therefore, laparotomy should be the recommended approach for cervical cancer patients.

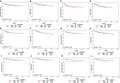

## 1 INTRODUCTION

As the fourth most frequently diagnosed cancer and the fourth leading cause of cancer‐related death, cervical cancer (CC) accounted for 570,000 new cases and 311,000 deaths in 2018 worldwide.^1^ In China, CC is responsible for 18.4% of cancer‐related deaths in women.^2^ Radical hysterectomy with bilateral pelvic lymph node dissection via minimally invasive surgery (MIS) or laparotomy has been considered the standard treatment for early stage CC for decades.^3^ However, the effect of the surgical approach on survival outcomes remains controversial.

MIS has consistently been shown to produce similar survival outcomes to laparotomy group, but results in shorter hospital stays and a lower risk of operative morbidity.^4^, ^5^, ^6^, ^7^, ^8^ Nevertheless, a phase III randomized controlled trial, “Laparoscopic Approach to Carcinoma of Cervix (LACC)”, identified an association of MIS with inferior disease‐free survival (DFS) and overall survival (OS) to open surgery.^9^ This trial triggered extensive discussion in clinical practice and prompted many new studies. Kim et al^10^ concluded that MIS was linked to higher recurrence rates than laparotomy in patients with stage IB1‐IIA2 CC, but was not a poor prognostic factor for patients with a tumor size ≤ 2 cm diagnosed with stage IB1 CC. In contrast, Cusimano et al^11^ reported poorer survival outcomes of MIS in the stage IB cohort, whereas no difference between MIS and laparotomy was identified in patients with other stages. Notably, the short‐term benefits of MIS had also been questioned. According to a recent study by a Chinese group, MIS is associated with a higher risk of major surgical complications than open surgery.^12^ Despite the many disputable findings on MIS, an analysis of possible contributing factors, such as surgeon proficiency, different medical circumstances among countries, etc, is lacking. Moreover, subgroup analyses of intermediate/high‐risk factors, such as histology, deep stromal invasion (DSI) and lymphovascular invasion (LVSI), are still lacking. Thus, additional solid evidence, particularly from multicenter larger cohort studies, is urgently needed to evaluate the effect of the surgical approach on survival outcomes.

To our knowledge, the largest sample size analyzed in this research area to date was 2461 in a study conducted in the United States.^13^ However, the evidence from populous Asian countries, such as China, particularly studies with a large sample size in high‐volume Chinese hospitals, is still lacking. Therefore, in the present study, we conducted a retrospective real‐world matched cohort study in four tertiary hospitals in China. We aimed to compare the survival outcomes of MIS and laparotomy and perform a comprehensive subgroup analysis of different combinations of various risk factors; we also aimed to assess the surgery trends and the effect of surgeon proficiency on survival outcomes. Moreover, we also determined the prognostic factors for RFS (recurrence‐free survival) and OS in our study population. Our study is the first to investigate the effect of the surgical approach in combination with multiple risk factors on survival outcomes in the largest sample size analyzed to date, which will provide additional evidence supporting the findings from previous studies and extend our understanding of surgical approach‐related survival outcomes in the real‐world setting.

## 2 MATERIALS AND METHODS

This retrospective multicenter cohort study was approved by the Institutional Ethics Committee of Fudan University Obstetrics and Gynecology Hospital (2019‐87). This study was registered in the Chinese Clinical Trial Registry (ChiCTR1900028702).

### 2.1 Study population

We identified patients with 2009 International Federation of Gynecology and Obstetrics (FIGO) stage IA1 (LVSI) to IIA1 CC who underwent radical hysterectomy according to the classification proposed by Querleu and Morrow^14^ from January 2006 to December 2017 in four tertiary hospitals as study population. Patients received type B or C radical hysterectomy based on different stages in accordance with the National Comprehensive Cancer Network (NCCN) guidelines at the time.^15^, ^16^ The exclusion criteria were as follows: (1) pregnant women, (2) <18 years old, (3) patients who received neoadjuvant therapy, (4) had a preexisting history of chemotherapy or radiotherapy for other conditions, (5) had a prior malignancy, (6) had an unclear lymphadenectomy status, (7) converted to laparotomy during the operation, and (8) had incomplete medical records or follow‐up data. In total, 3986 patients were enrolled in our study. 813 (20.4%) patients underwent laparotomy and 3173 (79.6%) received MIS. Patients who received robot‐assisted radical hysterectomy (RRH) or laparoscopic‐assisted radical vaginal hysterectomy (LARVH) were categorized into the MIS group for the intention‐to‐treat analysis. Of the 3173 patients who underwent MIS, 2956 (74.2%) received laparoscopic radical hysterectomy (LRH), 214 (5.4%) received RRH and 3 patients received LARVH.

### 2.2 Data collection

The retrospective data were obtained from four tertiary hospitals in Shanghai, China. All these hospitals were obstetrics and gynecology hospitals affiliated with a university. All medical records were reviewed simultaneously by three experts and independently checked by two experts to ensure the accuracy.

According to the NCCN guideline, preoperative workup for patients with suspicious symptoms includes history, physical examination, cervical cytologic screening, blood routine test (including platelets), liver and renal function, ECG, and imaging examinations. Radiologic imaging includes chest X‐ray, pelvic CT/MRI, or combined PET‐CT as indicated.^17^, ^18^ Cone biopsy is used if the cervical biopsy is inadequate to define invasiveness or if accurate assessment of microinvasive disease is required. When patients were older than 60, echocardiography, pulmonary function test and urodynamic test are also needed.

All patients underwent modified radical hysterectomy or radical hysterectomy with bilateral pelvic lymphadenectomy with or without para‐aortic lymphadenectomy according to NCCN guidelines. Before 2013, patients with IA1 (LVSI) received type B radical hysterectomy and patients with stage IA2‐IIA1 received type C radical hysterectomy. However, during 2013‐2017, patients with IA1 (LVSI) and IA2 underwent type B surgery, while those with IB1‐IIA1 underwent type C surgery. We began sentinel lymph node mapping in October 2016 by injecting 2‐4 ml methylene blue (MB) into the cervix (mainly at 2, 4, 8, and 10 o'clock position) before surgery. All blue nodes were considered sentinel nodes through intraoperative direct inspection. At out institution, we have used the uterine manipulator (RUMI, CooperSurgical, Inc, Trumbull, CT) during 2005‐2017 and have strictly followed the tumor‐free principle during surgical procedure. For type B radical hysterectomy, the vagina is transected such that a 2‐cm upper vaginal margin is included with the surgical specimen. For type C radical hysterectomy, the upper 1/4‐1/3 of vagina should be included. Surgeon characteristics were derived from the physician database of Fudan University Obstetrics and Gynecology Hospital. Surgeon proficiency was classified as skilled (≥50) and unskilled (<50) group according to the number of radical hysterectomies performed by the patient's surgeon in the 1 year prior to the patient's surgery year. These definitions ensured that surgeon proficiency could dynamically change over time.^19^

Patients were treated with adjuvant treatment after radical hysterectomy when they met one of the following two criteria: a) patients who presented any one of several high‐risk factors (positive surgical margin, parametrial involvement, and lymph node (LN) metastasis) and b) Sedlis et al^20^ criteria were satisfied for intermediate‐risk factors (tumor size, LVSI, and DSI). After hospital discharge, patients received regular follow‐up in accordance with the NCCN guidelines.^3^ The median follow‐up time was 90 (18‐162) months.

### 2.3 Variables and outcomes

All 18 variables were categorized into 6 clinical, 5 surgical and 7 pathological variables (Table S1). Lymph node metastasis was classified as no metastases, pelvic LNs common iliac LNs and para‐aortic LNs. If metastases were observed in two or more locations, then the furthest LNs station will be marked. For example, the patients with positive para‐aortic LNs and pelvic LN metastases will be classified as positive para‐aortic LNs.

The primary outcomes were RFS and OS, including 2‐ and 5‐year rates. RFS was defined as the interval from the initial CC diagnosis to the first finding of any recurrence or last follow‐up. OS was defined as the interval from the initial diagnosis to the CC‐related death or last follow‐up. Patients who failed to reach the survival events at the last follow‐up were censored. Local recurrences were defined by pathologic proof of cancer in the vagina/cervix which were confined to the pelvis or an imaging study showing regrowth of tumor or enlargement of any pelvic lymph node. Distant recurrences were also defined by pathologic, cytologic, or radiologic evidence. Any recurrence out of the pelvis, including peritoneal spread, involvement of supraclavicular lymph nodes, lung, liver, bone, brain, etc The definition of local or distant recurrence was determined by the lesions detected at the time of first relapse after a complete workup.

### 2.4 Statistical analysis

Continuous variables were reported as medians with interquartile ranges (IQRs) or means with standard deviations (SDs). Categorical variables were reported as number and proportions. We used Student's t‐test to compare continuous variables and Fisher's exact test or the χ^2^ test to compare categorical variables. The collinearity of all variables was evaluated using correlation matrices, and no significant interaction was identified. The Kaplan‐Meier method with the log‐rank test was used to compare survival outcomes. The associations of variables with RFS and OS were evaluated using Cox proportional hazards regression models. Hazard ratios (HRs) were presented with 95% confidence intervals (CIs).

Propensity score matching (PSM) was performed to reduce bias, according to the variables of FIGO stage, age, adjuvant treatment, parametrial involvement, LN metastasis, surgical margin, tumor size, histology, LVSI, and DSI. Matching was assessed by calculating the propensity scores before and after matching, *P*‐values > 0.05 indicate the success of matching.

Sensitivity analyses included propensity score weighting (PSW) and multivariate Cox regression analyses. In PSW, we set the weight of the laparotomy group to 1/propensity score and the weight of the MIS to 1/1 – propensity score.^21^ Absolute standardized differences less than 10% among variables indicated successful weighting. A multivariate Cox regression model was adjusted for same variables as in PSM.

Matching was performed in the stage IA1 (LVSI)‐IIA1 cohort, stage IB1 patient cohort, adjuvant treatment cohort, no‐adjuvant‐treatment cohort and each subgroup analysis for different combinations of various risk factors.

The statistical software packages used for analyses were SPSS (version 21.0; SPSS Inc, Chicago, IL, USA) and R 3.4.3(Vienna, Austria; http://www.R‐project.org/). All tests were two‐sided, and *P* < .05 was considered statistically significant.

## 3 RESULTS

**FIGURE 1:**
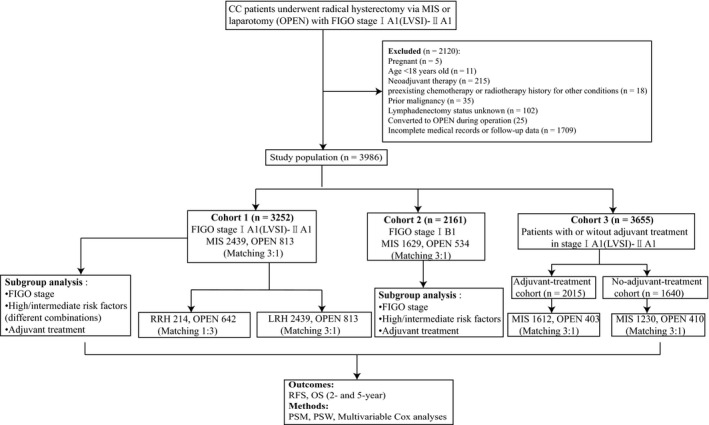
Flowchart of patients included in this study. Abbreviations: CC, cervical cancer; MIS, minimally invasive surgery; OPEN, open radical hysterectomy; RRH, robot‐assisted radical hysterectomy; LRH, laparoscopic radical hysterectomy

The study selection schematic and sample matching processes are presented in Figures 1. In total, 3986 patients with stage IA1 (LVSI)‐IIA1 CC who underwent MIS (3173) or laparotomy (813) from 2006 to 2017 were finally enrolled as the study population. After sample matching using PSM and PSW, three independent cohorts, 3252 (IA1 (LVSI)‐IIA1 CC), 2161 (IB1 CC), and 3655 (adjuvant treatment and no‐adjuvant‐treatment group) patients were studied.

### 3.1 Analysis of patients with stage IA1 (LVSI)‐IIA1 CC (cohort 1)

#### 3.1.1 Characteristics of the stage IA1 (LVSI)‐IIA1 CC cohort before and after matching

Characteristics of the MIS and laparotomy groups in the stage IA1 (LVSI)‐IIA1 cohort before and after PSM are presented in Table 1. After matching, all variables were similar between MIS and laparotomy group. The matched cohort contained 3252 patients, including 2439 who underwent MIS and 813 who underwent laparotomy. The median follow‐up period of 90 (18‐162) months, during which 178 patients (129 MIS and 49 laparotomy) died and 237 (181 MIS and 56 laparotomy) experienced recurrences. In terms of the recurrence site, local recurrence occurred in 143 (60.3%) patients, and distant recurrence occurred in 94 (39.7%) patients. Among the 143 patients who experienced local recurrence, 102 (71.3%) received MIS and 41 (28.7%) received laparotomy. In patients with distant recurrence, 79 (84%) underwent MIS and 15 (16%) underwent laparotomy. Moreover, lung metastasis (38, 40.4%) was the most common distant metastasis.

**TABLE 1:** Characteristics of stage A1(LVSI)‐ⅡA1 CC (cohort 1), before and after propensity‐score matching

Characteristics	Before matching (*n* = 3986)		*P*	After matching (*n* = 3252)		*P*
	MIS (*n* = 3173)	OPEN (*n* = 813)		MIS (*n* = 2439)	OPEN (*n* = 813)	
**Age**						
Mean ± SD	47.5 ± 9.6	47.9 ± 9.4	0.313	47.7 ± 9.8	47.9 ± 9.4	0.08
**FIGO stage (%)**						
1A1 (LVSI)	50 (1.6)	9 (1.1)	0.002	28 (1.1)	9 (1.1)	0.273
1A2	91 (2.9)	21 (2.6)		54 (2.2)	21 (2.6)	
1B1	2260 (71.2)	543 (66.8)		1683 (69)	543 (66.8)	
1B2	389 (12.3)	99 (12.2)		325 (13.3)	99 (12.2)	
2A1	383 (12.1)	141 (17.3)		349 (14.3)	141 (17.3)	
**Comorbidity (%)**						
No	2688 (84.7)	688 (84.6)	0.949	2049 (84)	688 (84.6)	0.677
Yes	485 (15.3)	125 (15.4)		390 (16)	125 (15.4)	
**Adjuvant treatment (%)**						
No	1404 (44.2)	410 (50.4)	0.002	1182 (48.5)	410 (50.4)	0.331
Yes	1769 (55.8)	403 (49.6)		1257 (51.5)	403 (49.6)	
**Tumor size, cm (%)**						
≤2	1235 (38.9)	305 (37.5)	0.137	932 (38.2)	305 (37.5)	0.223
(2,4]	1231 (38.8)	345 (42.4)		961 (39.4)	345 (42.4)	
>4	707 (22.3)	163 (20)		546 (22.4)	163 (20)	
**Histology (%)**						
SCC	2425 (76.4)	640 (78.7)	0.205	1924 (78.9)	640 (78.7)	0.995
AC	379 (11.9)	98 (12.1)		298 (12.2)	98 (12.1)	
AS	186 (5.9)	43 (5.3)		124 (5.1)	43 (5.3)	
Rare type	30 (0.9)	8 (1)		26 (1.1)	8 (1)	
Unknown	153 (4.8)	24 (3)		67 (2.7)	24 (3)	
**DSI (%)**						
Negative	852 (26.9)	181 (22.3)	0.026	557 (22.8)	181 (22.3)	0.919
<2/3	1005 (31.7)	268 (33)		808 (33.1)	268 (33)	
≥2/3	1316 (41.5)	364 (44.8)		1074 (44)	364 (44.8)	
**LVSI (%)**						
No	1859 (57.6)	501 (61.6)	0.116	1497 (61.4)	501 (61.6)	0.901
Yes	1314 (41.4)	312 (38.4)		942 (38.6)	312 (38.4)	
**Surgical margin (%)**						
No	2951 (93)	759 (93.4)	0.722	2281 (93.5)	759 (93.4)	0.87
Yes	222 (7)	54 (6.6)		158 (6.5)	54 (6.6)	
**Parametrial invasion (%)**						
No	3024 (95.3)	764 (94)	0.119	2300 (94.3)	764 (94)	0.729
Yes	149 (4.7)	49 (6)		139 (5.7)	49 (6)	
**LN metastasis (%)**						
No	2659 (83.8)	647 (79.6)	0.004	1982 (81.3)	647 (79.6)	0.292
Yes	514 (16.2)	166 (20.4)		457 (18.7)	166 (20.4)	
**Metastasis site (%)**						
No	2659 (83.8)	647 (79.6)	0.034	1982 (81.3)	647 (79.6)	0.607
Pelvic LN	386 (12.2)	129 (15.9)		341 (14)	129 (15.9)	
Common iliac LN	106 (3.3)	31 (3.8)		95 (3.9)	31 (3.8)	
Para‐aortic LN	22 (0.7)	6 (0.7)		21 (0.9)	6 (0.7)	

Abbreviations: AC, adenocarcinoma; AS, adenosquamous carcinoma; CC, cervical cancer; DSI, deep stromal invasion; FIGO, International Federation of Gynecology and Obstetrics; LN, lymph node; LVSI, lymphovascular space incision; MIS, minimally invasive surgery; OPEN, open radical hysterectomy; SCC, squamous cell carcinoma; SD, standard deviation.

#### 3.1.2 Analysis in matched patients with stage IA1 (LVSI)‐IIA1 CC

**FIGURE 2:**
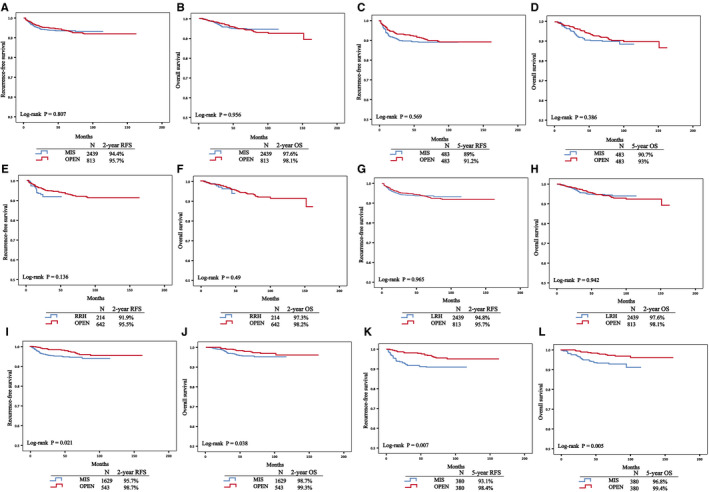
Survival outcome comparisons for matched patients. FIGO stage A1(LVSI)‐ⅡA1 cohort (A‐H); FIGO stage ⅠB1cohort (I‐L). Recurrence‐free survival (A, C, E, G, I, K); Overall survival (B, D, F, H, J, L). 2‐year (A, B, E‐H, I, J); 5‐year (C, D, K, L). Abbreviations: MIS, minimally invasive surgery; OPEN, open radical hysterectomy; RRH, robot‐assisted radical hysterectomy; LRH, laparoscopic radical hysterectomy

In the matched stage IA1 (LVSI)‐IIA1 CC cohort, the MIS and laparotomy groups showed similar 2‐year or 5‐year RFS and OS (2‐year RFS, 1.04 [0.76, 1.42], *P* = .807; 2‐year OS, 0.99 [0.70, 1.41], *P* = .956; 5‐year RFS, 1.12 [0.76, 1.65], *P* = .570; 5‐year OS, 1.20 [0.79, 1.83], *P* = .387) in all three analyses, including PSM (Figures 2A‐D), PSW (Figure S1A‐D) and multivariate Cox regression analyses. Additionally, we divided the MIS group into the robot‐assisted radical hysterectomy (RRH) and laparoscopic radical hysterectomy (LRH) (Figures 1E‐H). After matching (Table S2), no differences were observed in RFS and OS neither between the RRH and laparotomy groups (RFS, 1.546 [0.867, 2.755], *P* = .14; OS, 1.326 [0.593, 2.959], *P* = .492) or between the LRH and laparotomy groups (RFS, 0.993 [0.725, 1.361], *P* = .965; OS, 1.013 [0.714, 1.437], *P* = .942). Nevertheless, we wondered whether some differences in survival outcomes might exist between the MIS and laparotomy groups when patients were stratified by subgroups. Therefore, we performed the subgroup analyses described below.

#### 3.1.3 Subgroup analysis in matched patients with stage IA1 (LVSI)‐IIA1 CC

**FIGURE 3:**
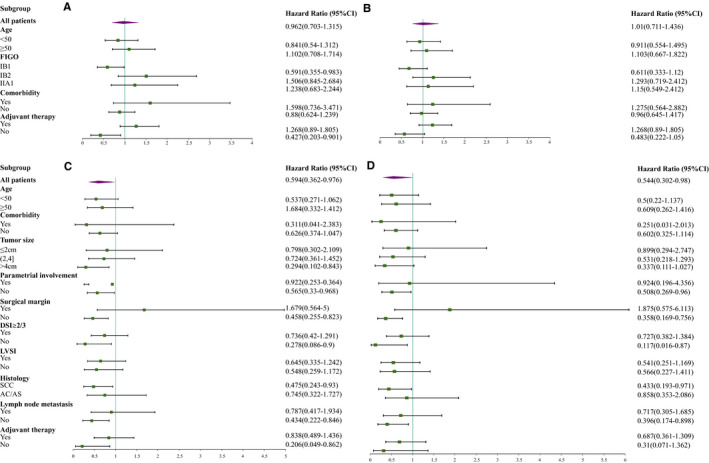
Subgroup analysis. FIGO stage A1(LVSI)‐ⅡA1 cohort (A‐B); FIGO stage B1cohort (C‐D). Recurrence‐free survival (A, C); Overall survival (B, D). Abbreviations: FIGO, International Federation of Gynecology and Obstetrics; SCC, squamous cell carcinoma; AC, adenocarcinoma; AS, adenosquamous carcinoma; DSI, deep stromal invasion; LVSI, lymphovascular space incision

A subgroup analysis was then performed in the matched stage IA1 (LVSI)‐IIA1 cohort according to age, FIGO stage, comorbidity, and adjuvant treatment (Figures 3A and B). We identified poorer RFS (HR, 1.691 [1.017, 2.814], *P* = .04) for patients with stage IB1 who underwent MIS, whereas the OS was similar between patients who underwent MIS and laparotomy. In terms of adjuvant treatment, the MIS group exhibited a worse RFS (HR 2.34, *P* = .02) and similar OS to the laparotomy group for patients treated without adjuvant treatment, while the RFS and OS were similar between the two groups for patients treated with adjuvant treatment.

Additionally, we also performed a more comprehensive subgroup analysis according to intermediated/high‐risk factors (Table S3). Three high‐risk factors (surgical margin, LN metastasis and parametrial involvement) and four intermediate‐risk factors (tumor size, DSI, LVSI and histology) formed 11 different combinations of subgroups. Matching was performed in each subgroup. Interestingly, we identified a poorer RFS of MIS for patients with any one factor among (>2 cm, LVSI, DSI, non‐SCC (squamous cell carcinoma)). However, no difference between MIS and laparotomy were found for patients with more risk factors.

Based on these findings, MIS might result in poor survival outcomes in patients with stage IB1, without adjuvant treatment and even less risk factors. Therefore, we focused on the stage IB1 cohort and adjuvant‐treatment cohort in the subsequent study to confirm the robustness of our findings.

### 3.2 Analysis of patients with stage IB1 CC (cohort 2)

#### 3.2.1 Characteristics of the stage IB1 CC cohort before and after matching

Because higher recurrence risk was observed for MIS in the stage IB1 subgroup, we therefore performed a deep analysis (Table 2). Before matching, the proportion of patients treated with adjuvant treatment was greater in the MIS group, while LN metastasis was less common in the MIS group. After matching, no significant differences in all variables were observed between the two groups. In the matched stage IB1 cohort, 2172 patients were analyzed (1629 MIS and 543 laparotomy). One hundred twenty‐three recurrences (104 MIS and 19 laparotomy) and 83 deaths (69 MIS and 14 laparotomy) occurred.

**TABLE 2:** Characteristics of stage B1 CC (cohort 2), before and after propensity‐score matching

Characteristics	Before matching (*n* = 2803)		*P*	After matching (*n* = 2172)		*P*
	MIS (*n* = 2260)	OPEN (*n* = 543)		MIS (*n* = 1629)	OPEN (*n* = 543)	
**Age**						
Mean ± SD	47.1 ± 9.5	47.4 ± 9.4	0.380	47.3 ± 9.4	47.4 ± 9.4	0.577
**Comorbidity (%)**						
No	1911 (84.6)	468 (86.2)	0.341	1368(84)	468(86.2)	0.217
Yes	349 (15.4)	75 (13.8)		261(16)	75(13.8)	
**Adjuvant treatment (%)**						
No	1101 (48.7)	301 (55.4)	0.005	881(54.1)	301(55.4)	0.584
Yes	1159 (51.3)	242 (44.6)		748(45.9)	242(44.6)	
**Tumor size, cm (%)**						
≤2	1026(45.4)	236(43.5)	0.450	699(42.9)	236(43.5)	0.973
(2,4]	894(39.6)	214(39.4)		650(39.9)	214(39.4)	
>4	340(15)	93(17.1)		280(17.2)	93(17.1)	
**Histology (%)**						
SCC	1668(73.8)	420(77.3)	0.208	1229(75.4)	420(77.3)	0.467
AC	316(14)	71(13.1)		249(15.3)	71(13.1)	
AS	137(6.1)	29(5.3)		98(6)	29(5.3)	
Rare type	24(1.1)	7(1.3)		12(0.7)	7(1.3)	
Unknown	115(5.1)	16(2.9)		41(2.5)	16(2.9)	
**DSI (%)**						
Negative	651(28.8)	133(24.5)	0.131	381(23.4)	133(24.5)	0.814
<2/3	800(35.4)	202(37.2)		628(38.6)	202(37.2)	
≥2/3	809(35.8)	208(38.3)		620(38.1)	208(38.3)	
**LVSI (%)**						
No	1365(60.4)	345(63.5)	0.178	1029(63.2)	345(63.5)	0.877
Yes	895(39.6)	198(36.5)		600(36.8)	198(36.5)	
**Surgical margin (%)**						
No	2143 (94.8)	517 (95.2)	0.712	1549(95.1)	517(95.2)	0.908
Yes	117 (5.2)	26 (4.8)		80(4.9)	26(4.8)	
**Parametrial invasion (%)**						
No	2189 (96.9)	526 (96.9)	0.99	1570(96.4)	526(96.9)	0.59
Yes	71 (3.1)	17 (3.1)		59(3.6)	17(3.1)	
**LN metastasis (%)**						
No	1967(87)	450(82.9)	0.11	1359(83.4)	450(82.9)	0.765
Yes	293(13)	93(17.1)		270(16.6)	93(17.1)	
**Metastasis site (%)**						
No	1967(87)	450(82.9)	0.076	1359(83.4)	450(82.9)	0.948
Pelvic LN	228(10.1)	74(13.6)		211(13)	74(13.6)	
Common iliac LN	51(2.3)	16(2.9)		47(2.9)	16(2.9)	
Para‐aortic LN	14(0.6)	3(0.6)		12(0.7)	3(0.6)	

Abbreviations: AC, adenocarcinoma; AS, adenosquamous carcinoma; CC, cervical cancer; DSI, deep stromal invasion; FIGO, International Federation of Gynecology and Obstetrics; LN, lymph nodeLVSI, lymphovascular space incision; MIS, minimally invasive surgery; OPEN, open radical hysterectomy; SCC, squamous cell carcinoma; SD, standard deviation.

#### 3.2.2 Deep analysis in matched patients with stage IB1 CC

In the matched stage IB1 cohort (Figure 2I‐L), MIS resulted in a poorer 2‐year (RFS, HR 1.81 [1.085, 3.003], *P* = .023; OS, HR 1.87 [1.025, 3.401], *P* = .041) and 5‐year RFS and OS (RFS, HR 2.17 [1.212, 3.891], *P* = .009; OS, HR 2.57 [1.294, 5.102], *P* = .007).

PSW and multivariate Cox regression analyses were performed to test the robustness of our results. In the propensity‐weighted cohort, all covariates were balanced, with SDs less than 10% (Figure S2) and *P*‐values > 0.05. Kaplan‐Meier curves showed inferior RFS and OS for the MIS group compared to the laparotomy group (RFS, HR 1.6, *P* = .025; OS, HR 1.79, *P* = .018) (Figure S1E‐H). The multivariate Cox regression analysis yielded consistent results (RFS, HR 1.65, *P* = .048; OS HR 1.88, *P* = .037). Altogether, these three methods reveal a higher risk of recurrence and death in patients with stage IB1 CC who underwent MIS than in the laparotomy group.

#### 3.2.3 Subgroup analysis in matched patients with stage IB1 CC

Next, we performed a subgroup analysis of the matched stage IB1 CC cohort (Figures 3C and D). The MIS group showed worse RFS and OS than the laparotomy group in the following subgroups: a) negative parametrial involvement (RFS, *P* = .03; OS, *P* = .04); b) negative surgical margin (RFS, *P* = .005; OS, *P* = .006); c) negative LN metastasis (RFS, *P* = .03; OS, *P* = .04) and d) DSI < 2/3 (RFS, *P* = .04; OS, *P* = .03). In terms of tumor size, patients with tumor size > 4 cm showed an inferior RFS (HR 3.58, *P* = .01) in MIS, whereas the OS was similar between two groups.

In the analysis of the histological type, poorer RFS and OS were observed for patients with SCC in the MIS group (RFS, HR 2.22, *P* = .02; OS, HR 2.31, *P* = .04), whereas similar RFS and OS were observed in patients with adenocarcinoma (AC) or adenosquamous carcinoma (AS) between two groups. In addition, consistent with the results obtained for the stage IA1 (LVSI)‐IIA1 cohort, a higher recurrence risk was observed for patients in the no‐adjuvant‐treatment group who received MIS (RFS, HR 5.85, *P* = .006), whereas no significant differences between MIS and laparotomy were observed in the adjuvant‐treatment group.

In conclusion, MIS resulted in poor survival outcomes in many subgroups of the stage IB1 CC cohort, such as SCC histology, no‐adjuvant‐treatment group and even certain low‐risk subgroups.

### 3.3 Analysis of surgical approach‐related survival outcomes in patients treated with or without adjuvant treatment (cohort 3)

Because a higher recurrence risk was observed in patients who were not treated with adjuvant treatment in the matched stage IA1 (LVSI)‐IIA1 and stage IB1 cohorts who underwent MIS, we therefore focused on patients treated with or without adjuvant treatment and performed a deep analysis. Before matching (Table S4), 2172 patients, predominantly with stage IB1‐IIA1 CC (2138, 98.4%), received adjuvant treatment (1769 MIS and 403 laparotomy) while 1814 (1404 MIS and 410 laparotomy) did not receive further treatment. Concurrent chemoradiation therapy (CCRT) (1427) was the most common type, followed by chemotherapy and radiotherapy. Patients in the adjuvant‐treatment group had more risk factors than patients in the no‐adjuvant‐treatment group.

In matched adjuvant‐treatment cohort, all variables other than LN metastasis were similar between the MIS and laparotomy groups (Table S5). In the survival analysis, neither PSM nor multivariate analyses identified differences in RFS and OS between the MIS and laparotomy groups (RFS, *P* = .051; OS, *P* = .087) (Figure S3).

In the matched no‐adjuvant‐treatment cohort, with the exception of the FIGO stage, tumor size and DSI, all variables were similar between the two groups (Table S5). Consistent with the result of subgroup analyses of both the stage IA1 (LVSI)‐IIA1 and stage IB1 cohorts, both PSM and multivariate analyses revealed a worse RFS for the MIS group than the laparotomy group (HR 2.24, *P* = .033) in the no‐adjuvant‐treatment cohort, whereas the OS was similar in the MIS and laparotomy groups (Figure S3).

Therefore, we concluded that patients with stage IA1 (LVSI)‐IIA1 CC who received MIS had a higher recurrence risk than patients who received laparotomy in the no‐adjuvant‐treatment cohort. However, in patients treated with adjuvant treatment, the survival outcomes of MIS and laparotomy were similar.

### 3.4 Analysis of temporal trends and surgeon proficiency in the surgical approach

The trends of MIS and laparotomy between 2003 and 2017 are shown in Figure S4. The use of MIS significantly increased from 0% in 2003 to 99.42% in 2017, and MIS became the dominant surgery approach used since 2010 (52.79%).

We also analyze the effect of surgeon proficiency (skilled/unskilled) on survival outcomes in the MIS and laparotomy groups. Significant differences in vital variables were not identified between skilled and unskilled groups (Table S6). In the survival analysis, poorer RFS and OS were observed in the unskilled group (RFS, HR 6.274, *P* < .01; OS, HR 5.195, *P* < .01). When stratified according to the surgical approach, we noticed an inferior RFS and OS of patients who underwent MIS in the unskilled group (RFS, HR 7.346, *P* < .01; OS, HR 6.161, *P* < .01). However, differences were not observed in the survival of patients who received laparotomy between the skilled and unskilled groups (Figure S5). Thus, inadequate surgeon proficiency strongly correlated with poor RFS and OS in patients who underwent MIS, but not in patients who underwent laparotomy.

### 3.5 Univariate and multivariate analyses of patients with stage IA1 (LVSI)‐IIA1 CC

We next performed univariate and multivariate Cox analyses of the stage IA1 (LVSI)‐IIA1 CC to investigate the comprehensive prognostic factors for RFS or OS (Table 3). According to the univariate analysis, with the exception of age, comorbidity and surgical approach, all variables showed *P* values < 0.05 for both RFS and OS. LN metastasis, parametrial involvement, DSI, histology and FIGO stage were identified as independent prognostic factors for a poor RFS in the multivariate analysis. The FIGO stage, LN metastasis, surgical margin, DSI, and histology were identified as independent prognostic factors for a poor OS.

**TABLE 3:** Factors associated with recurrence‐free survival and overall survival in stage A1(LVSI)‐ⅡA1 CC patients

Characteristics	No.	RFS				OS			
		Univariate		Multivariate		Univariate		Multivariate	
		HR (95%CI)	*P*	HR (95%CI)	*P*	HR (95%CI)	*P*	HR (95%CI)	*P*
Age									
<50	2379	1	0.072			1	0.076		
≥50	1607	1.265[0.979,1.634]				1.307[0.972,1.757]			
FIGO (%)			<0.001		<0.001		<0.001		<0.001
1A1 (LVSI)	59	1		1		1		1	
1A2	112	0.338[0.056,2.023]		0.517[0.086,3.119]		0.518[0.073,3.679]		0.823[0.114,5.925]	
1B1	2803	0.827[0.263,2.601]		0.693[0.218,2.2]		0.821[0.202,3.339]		0.67[0.164,2.741]	
1B2	488	2.365[0.741,7.549]		1.45[0.45,4.674]		3.46[0.845,14.175]		2.091[0.508,8.609]	
2A1	524	1.984[0.619,6.356]		1.119[0.345,3.629]		2.059[0.494,8.572]		1.138[0.271,4.778]	
Comorbidity (%)			0.658				0.275		
No	3376	1				1			
Yes	610	1.087[0.752,1.57]				1.241[0.842,1.829]			
Surgery approach (%)			0.89				0.936		
MIS	3173	1				1			
OPEN	813	1.022[0.753,1.385]				1.014[0.722,1.424]			
Adjuvant treatment (%)			<0.001				<0.001		
No	1814	1				1			
Yes	2172	2.309[1.734,3.076]				2.454[1.754,3.432]			
Tumor size, cm (%)			<0.001				<0.001		
≤2	1540	1				1			
(2,4]	1576	1.978[1.399,2.797]				1.909[1.27,2.87]			
>4	870	3.737[2.637,5.295]				4.366[2.922,6.524]			
Histology (%)			<0.001		<0.001		<0.001		<0.001
SCC	3065	1		1		1		1	
AC	477	1.6[1.138,2.251]		1.763[1.248,2.489]		1.904[1.308,2.772]		2.078[1.423,3.035]	
AS	229	1.399[0.849,2.303]]		1.347[0.816,2.224]		1.534[0.866,2.718]		1.454[0.818,2.584]	
Rare type	38	5.039[2.575,9.86]		5.108[2.602,10.028]		4.138[1.691,10.127]		4.896[1.991,12.038]	
Unknown	177	0.112[0.016,0.797]		0.321[0.044,2.366]		0.189[0.026,1.352]		0.638[0.085,4.765]	
DSI (%)			<0.001		<0.001		<0.001		<0.001
Negative	1033	1		1		1		1	
<2/3	1273	1.922[1.141,3.238]		1.519[0.891,2.591]		2.483[1.261,4.889]		2.027[1.01,4.068]	
≥2/3	1680	5.346[3.363,8.498]		3.12[1.915,5.085]		7.315[3.954,13.533]		4.173[2.184,7.973]	
LVSI (%)			<0.001				<0.001		
No	2360	1				1			
Yes	1626	2.049[1.583,2.65]				2.289[1.697,3.087]			
Surgical margin (%)			<0.001		0.061		<0.001		0.015
No	3710	1		1		1		1	
Yes	276	2.301[1.593,3.325]		1.449[0.982,2.137]		2.503[1.65,3.796]		1.705[1.109,2.623]	
Parametrial involvement (%)			<0.001		0.028		<0.001		
No	3788	1		1		1			
Yes	198	3.484[2.421,5.007]		1.559[1.05,2.316]		3.25[2.114,4.998]			
LN metastasis (%)			<0.001		<0.001		<0.001		<0.001
No	3306	1		1		1		1	
Yes	680	3.282[2.527,4.262]		1.928[1.45,2.565]		3.64[2.7,4.908]		2.241[1.635,3.071]	

Abbreviations: AC, adenocarcinoma; AS, adenosquamous carcinoma; CC, cervical cancer; DSI, deep stromal invasion; FIGO, International Federation of Gynecology and Obstetrics; LN, lymph node; LVSI, lymphovascular space incision; MIS, minimally invasive surgery; OPEN, open radical hysterectomy; SCC, squamous cell carcinoma; SD, standard deviation.

## 4 DISCUSSION

In this study, we evaluated the survival outcomes of 3986 patients with FIGO stage IA1 (LVSI)‐IIA1 CC who underwent radical hysterectomy via MIS and laparotomy. (1) The 2‐ and 5‐year RFS (2‐year, hazard ratio [HR], 1.81; 95% confidence interval [CI], 1.09‐3.0; 5‐year, HR, 2.17; 95% CI, 1.21‐3.89) or OS (2‐year, HR, 1.87; 95% CI, 1.03‐3.40; 5‐year, HR, 2.57; 95% CI, 1.29‐5.10) were significantly worse for MIS in patients with stage I B1, but not the cohort overall (2‐year RFS, HR, 1.04; 95% CI, 0.76‐1.42; 2‐year OS, HR, 0.99; 95% CI, 0.70‐1.41; 5‐year RFS, HR, 1.12; 95% CI, 0.76‐1.65; 5‐year OS, HR, 1.20; 95% CI, 0.79‐1.83) or other stages (2) In a subgroup analysis, MIS exhibited poorer survival in many population subsets, even in patients with less risk factors, such as patients with squamous cell carcinoma, negative for parametrial involvement, with negative surgical margins, negative for lymph node metastasis, and deep stromal invasion < 2/3. (3) In the cohort treated with or without adjuvant treatment, MIS resulted in a worse RFS than laparotomy in patients treated without adjuvant treatment, whereas no differences in RFS and OS were observed in patients treated with adjuvant treatment. (4) Inadequate surgeon proficiency strongly correlated with poor RFS and OS in patients who underwent MIS than in patients who underwent laparotomy.

After a decade of widespread acceptance of MIS, the results of the LACC upended the previous consensus. However, some results from the trial remain controversial, including early termination, inadequate pathological review, and follow‐up data, the dominant RRH trend in the United States,^22^ and the lack of an assessment of surgeon proficiency. Based on recently emerging new evidence,^10^, ^11^, ^23^, ^24^, ^25^, ^26^, ^27^, ^28^, ^29^ with the exception of the findings reported by Doo et al^29^ and Alfonzo et al^28^ which provide no evidence that MIS results in an inferior survival outcome compared to laparotomy, most studies reported consistent results with the LACC. Nevertheless, the majority of those studies were small sample size and lack of a comprehensive subgroup analysis of crucial pathological factors. Therefore, we performed this multicenter matched study to provide additional evidence in support of previous studies.

Consistent with the findings reported by Cusimano et al^11^ we observed inferior RFS and OS for patients with stage IB1 CC who underwent MIS compared to the laparotomy group. These findings were verified using various methods, including PSM, PSW and multivariate Cox regression analyses. In addition, by performing a subgroup analysis, we observed poorer survival outcomes for patients who received MIS in various population subgroups, including the SCC subgroup and certain low‐risk subgroups, such as patients with negative parametrial involvement, negative surgical margins, negative LN metastasis, DSI < 2/3, etc Patients with CC presenting with high/intermediate risk factors should receive adjuvant treatment, according to the NCCN guidelines. Therefore, the explanation for the lack of differences between MIS and laparotomy in patients with high‐risk factors may potentially be the adjuvant treatment, which might mask the poor performance of MIS. Notably, in terms of adjuvant treatment, our results indeed showed similar RFS and OS were observed in patients treated with adjuvant treatment in the MIS and laparotomy groups, but inferior RFS was observed in patients treated without adjuvant treatment in the MIS group compared to the laparotomy group. Admittedly, these results should be further confirmed in future prospective studies with large sample sizes.

In the current study, a higher risk of recurrence and death was observed in various population subgroups who underwent MIS. The possible explanations include a) the use of a uterine manipulator might crush the cervical tumor and lead to dissemination, (b) the intracorporeal colpotomy might cause tumor implantation metastasis in the pelvic cavity in the abdomen, and (c) CO_2_ levels in the pneumoperitoneum might adversely affect the survival outcome by accelerating tumor growth and spread.^30^ (d) Although many previous studies showed short‐term benefits of MIS, including shorter hospital stays and fewer infections, etc, some emerging new evidence suggested an association between MIS and a higher risk of major surgical complications than laparotomy.^12^ Despite these possible explanations, based on our findings, inadequate surgeon proficiency might also exert an adverse effect on survival outcomes. Patients who underwent MIS performed by an unskilled surgeon displayed significantly worse RFS and OS than patients who received an operation by a skilled surgeon, while no differences were observed in patients who underwent laparotomy. Thus, inadequate surgeon proficiency strongly correlated with poor RFS and OS in patients who underwent MIS, but not in patients who underwent laparotomy.

Consistent with previous studies,^10^ the MIS group showed worse RFS and OS than the laparotomy group in patients with SCC, whereas no difference was observed in patients without SCC. Adenocarcinoma, one of the most common types of non‐SCC tumors, is mainly a multifocal lesion that is difficult to detect when it occurs at a high level in the cervical canal, and it is known to have a worse prognosis than SCC. However, the aggressive nature of non‐SCC may mask the poor performance of MIS. In addition, due to the endophytic behavior, it is less likely to affect the tumor‐free principle of MIS. All these possibilities might explain the different performance of MIS and laparotomy in terms of patients with different histological types of CC.

Some limitations of our study are listed below. First, although we strictly adhered to the inclusion and exclusion criteria, bias might still exist due to the retrospective study design and large time span. Second, heterogeneity might exist among our four hospitals. Third, the relatively small number of patients with stage IA, IB2 and IIA1 CC limited our power to detect differences between MIS and laparotomy. Thus, stricter quality control measures and more evidence on those stages obtained with new pathology‐based FIGO criteria are needed. These limitations might be overcome in future prospective studies.

Notably, our study has several strengths. First, to our knowledge, this study is the first to perform a comprehensive subgroup analysis on different combinations of various intermediate/high‐risk factors, such as the histological type, DSI and LVSI. Second, this study is analyzed the largest multicenter cohort with the longest follow‐up reported to date for the survival outcomes based on real world evidence. Third, various matching methods were performed to reduce bias, including PSM, PSW and multivariate Cox analyses; the consistent results obtained using all methods confirmed the robustness. Finally, we separately compared RRH or LRH with laparotomy.

In conclusion, our four‐center matched cohort study identified an association of MIS with inferior RFS and OS compared to laparotomy in patients with stage IB1 CC and various population subgroups, including the SCC subgroup and certain low‐risk subgroups. Moreover, MIS resulted in a worse RFS in patients treated with adjuvant treatment, whereas no differences in RFS and OS were observed in patients treated without adjuvant treatment. Additionally, inadequate surgeon proficiency strongly correlated with poor RFS and OS in patients who underwent MIS, but not laparotomy. Considering the poor survival outcomes of MIS in various population subgroups, laparotomy might be the recommended approach for patients with CC.

## CONFLICT OF INTEREST

The authors report no conflict of interest.

## AUTHOR CONTRIBUTIONS

Conceptualization: Chenyan Guo, Junjun Qiu, Methodology: Chenyan Guo, Xiaoyan Tang, Yan Meng, Data acquisition: Chenyan Guo, Jingjing Guo, Xiaohong Lei, Xiaoyan Tang, Xuyin Zhang, Ying Zhang, Validation: Keqin Hua, Junjun Qiu; Writing‐original draft: Chenyan Guo; Writing‐review & editing: all authors; Supervision: Keqin Hua, Junjun Qiu.

## Supporting information


**Figure S1.**

**Figure S2.**

**Figure S3.**

**Figure S4.**

**Figure S5.**

**supinfo.**

## Data Availability

The data that support the findings of this study are available on request from the corresponding author. The data are not publicly available due to privacy or ethical restrictions.
