# Supplementary material for: Effect of the surgical approach on survival outcomes in patients undergoing radical hysterectomy for cervical cancer: A real‐world multicenter study of a large Chinese cohort from 2006 to 2017
Source: Cancer Med. 2020 Jul 6;9(16):5908–21. doi: 10.1002/cam4.3287 (PMC7433827; doi:10.1002/cam4.3287)
Supplement: Supplementary file 6 — supinfo. [file CAM4-9-5908-s006.docx]

**Supplementary appendix**

**Table S1**

Baseline characteristics of stageⅠA1(LVSI)-ⅡA1 CC cohort

| Characteristics | Number of patients (n=3986) |
| --- | --- |
| **Clinical variables** |  |
| **Age, years** | 47.6 (± 9.6) |
| **FIGO stage** |  |
| ⅠA1 (LVSI) | 59 (1.5) |
| ⅠA2 | 112 (2.8%) |
| ⅠB1 | 2803 (70.3%) |
| ⅠB2 | 488 (12.2%) |
| ⅡA1 | 524 (13.1%) |
| **Comorbidity** |  |
| Yes | 610 (15.3%) |
| No | 3376 (84.7%) |
| **HPV infection** |  |
| Yes | 1722 (43.2%) |
| 16/18 | 1000 (25.1%) |
| Non 16/18 | 722 (18.1%) |
| No | 580 (14.6%) |
| Unknown | 1684 (42.2%) |
| **SCCA** | 3.2 (± 5.3) |
| **Adjuvant treatment** |  |
| Yes | 2172 (54.5%) |
| Chemotherapy | 386 (9.7%) |
| Radiotherapy | 359 (9%) |
| CCRT | 1427 (35.8%) |
| No | 1814 (45.5%) |
| **Surgery related variables** |  |
| **Surgery approach** |  |
| MIS | 3173 (79.6%) |
| LRH | 2956 (74.2%) |
| RRH | 214 (5.4%) |
| LARVH | 3 (0.1%) |
| OPEN | 813 (20.4%) |
| **Operative time, min** | 200 (165, 246) |
| **Blood loss, ml** | 200 (150, 400) |
| **Transfusion** |  |
| Yes | 242 (6.1%) |
| No | 3744 (93.9%) |
| **LEEP** |  |
| Yes | 885 (22.2%) |
| No | 3101 (77.8%) |
| **Pathologic variables** |  |
| **Tumor size, cm** |  |
| ≤2 | 1540 (38.6%) |
| (2,4] | 1576 (39.5%) |
| >4 | 870 (21.8%) |
| **Histology** |  |
| SCC | 3065 (76.9%) |
| AC | 477 (12%) |
| AS | 229 (5.7%) |
| Rare type | 38 (1%) |
| Unknown | 177 (4.4%) |
| **DSI** |  |
| Negative | 1033 (25.9%) |
| <2/3 | 1273 (31.9%) |
| ≥2/3 | 1680 (42.1%) |
| **LVSI** |  |
| Yes | 1626 (40.8%) |
| No | 2360 (59.2%) |
| **Surgical margin** |  |
| Yes | 276 (6.9%) |
| No | 3710 (93.1%) |
| **Parametrial involvement** |  |
| Yes | 198 (5%) |
| No | 3788 (95%) |
| **LN metastasis** |  |
| Yes | 680 (17.1%) |
| Pelvic LNs | 515 (12.9%) |
| Common iliac LNs | 137 (3.4%) |
| Para-aortic LNs | 28 (0.7%) |
| No | 3306 (82.9%) |
| **Follow-up, months** | 90(18-162) |

**Table S2**

Characteristics of stage ⅠA1(LVSI)-ⅡA1 CC patients underwent RRH or LRH and laparotomy, after propensity-score matching

| Characteristics | After matching(n=856) | | |  | After matching(n=3252) | | |
| --- | --- | --- | --- | --- | --- | --- | --- |
|  | RRH  (n=214) | OPEN  (n=642) | P |  | LRH  (n=2439) | OPEN  (n=813) | P |
| **Age** |  |  | 0.85 |  |  |  | 0.418 |
| Mean ± SD | 47.2±9.4 | 47.3±9.5 |  |  | 47.6±9.6 | 48±9.4 |  |
| **FIGO stage (%)** |  |  | 0.103 |  |  |  | 0.057 |
| 1A1 (LVSI) | 2(0.9%) | 6(0.9%) |  |  | 26(1.1%) | 9(1.1%) |  |
| 1A2 | 3(1.4%) | 10(1.6%) |  |  | 58(2.4%) | 21(2.6%) |  |
| 1B1 | 149(69.6%) | 415(64.6%) |  |  | 1701(69.7%) | 543(66.8%) |  |
| 1B2 | 14(6.5%) | 87(13.6%) |  |  | 332(13.6%) | 99(12.2%) |  |
| 2A1 | 46(21.5%) | 124(19.3%) |  |  | 322(13.2%) | 141(17.3%) |  |
| **Comorbidity (%)** |  |  | 0.774 |  |  |  | 0.933 |
| No | 186(86.9%) | 553(86.1%) |  |  | 2067(84.7%) | 688(84.6%) |  |
| Yes | 28(13.1%) | 89(13.9%) |  |  | 372(15.3%) | 125(15.4%) |  |
| **Adjuvant therapy (%)** |  |  | 0.608 |  |  |  | 0.162 |
| No | 108(50.5%) | 311(48.4%) |  |  | 1161(47.6%) | 410(50.4%) |  |
| Yes | 106(49.5%) | 331(51.6%) |  |  | 1278(52.4%) | 403(49.6%) |  |
| **Tumor size, cm (%)** |  |  | 0.344 |  |  |  | 0.194 |
| ≤2 | 85(39.7%) | 227(35.4%) |  |  | 950(39%) | 305(%) |  |
| (2,4] | 83(38.8%) | 285(44.4%) |  |  | 951(39%) | 345(42.4%) |  |
| >4 | 46(21.5%) | 130(20.2%) |  |  | 538(22.1%) | 163(20%) |  |
| **Histology (%)** |  |  | 0.102 |  |  |  | 0.8 |
| SCC | 152(71%) | 486(75.7%) |  |  | 1918(78.6%) | 640(78.7%) |  |
| AC | 31(14.5%) | 82(12.8%) |  |  | 293(12%) | 98(12.1%) |  |
| AS | 13(6.1%) | 42(6.5%) |  |  | 147(6%) | 43(5.3%) |  |
| Rare type | 1(0.5%) | 8(1.2%) |  |  | 25(1%) | 8(1%) |  |
| Unknown | 17(7.9%) | 24(3.7%) |  |  | 56(2.3%) | 24(3%) |  |
| **DSI (%)** |  |  | 0.872 |  |  |  | 0.538 |
| Negative | 57(26.6%) | 160(24.9%) |  |  | 586(24%) | 181(22.3%) |  |
| <2/3 | 64(29.9%) | 200(31.2%) |  |  | 804(33%) | 268(33%) |  |
| ≥2/3 | 93(43.5%) | 282(43.9%) |  |  | 1049(43%) | 364(44.8%) |  |
| **LVSI (%)** |  |  | 0.904 |  |  |  | 0.868 |
| No | 126(58.9%) | 375(58.4%) |  |  | 1495(61.3%) | 501(61.6%) |  |
| Yes | 88(41.1%) | 267(41.6%) |  |  | 944(38.7%) | 312(38.4%) |  |
| **Surgical margin (%)** |  |  | 1 |  |  |  | 0.805 |
| No | 197(92.1%) | 591(92.1%) |  |  | 2283(93.6%) | 759(93.4%) |  |
| Yes | 17(7.9%) | 51(7.9%) |  |  | 156(6.4%) | 54(6.6%) |  |
| **Parametrial invasion (%)** |  |  | 0.786 |  |  |  | 0.663 |
| No | 204(95.3%) | 609(94.9%) |  |  | 2302(94.4%) | 764(94%) |  |
| Yes | 10(4.7%) | 33(5.1%) |  |  | 137(5.6%) | 49(6%) |  |
| **LN metastasis (%)** |  |  | 0.334 |  |  |  | 0.28 |
| No | 184(86%) | 534(83.2%) |  |  | 1983(81.3%) | 647(79.6%) |  |
| Yes | 30(14%) | 108(16.8%) |  |  | 456(18.7%) | 166(20.4%) |  |
| **Metastasis site (%)** |  |  | 0.754 |  |  |  | 0.736 |
| No | 184(86%) | 534(83.2%) |  |  | 1983(81.3%) | 647(79.6%) |  |
| Pelvic LN | 21(9.8%) | 80(12.5%) |  |  | 349(14.3%) | 129(15.9%) |  |
| Common iliac LN | 7(3.3%) | 23(3.6%) |  |  | 90(3.7%) | 31(3.8%) |  |
| Para-aortic LN | 2(0.9%) | 5(0.8%) |  |  | 17(0.7%) | 6(0.7%) |  |

**Table S3**

Subgroup analysis of different combinations of various high/intermediate risk factors, after matching

| Subgroup | No. | No. | RFS | |  | OS | |
| --- | --- | --- | --- | --- | --- | --- | --- |
|  | MIS | OPEN | HR (95%CI) | P |  | HR (95%CI) | P |
| **High-risk factors** | | | |  |  |  |  |
| Any 1 of 3 factors | 549 | 163 | 0.631 [0.376,1.06] | 0.08 |  | 0.769 [0.426,1.385] | 0.4 |
| Any 2 of 3 factors | 135 | 38 | 1.216 [0.542,2.729] | 0.6 |  | 1.2 [0.499,2.89] | 0.7 |
| All 3 factors | 22 | 10 | 1.17 [0.226,6.061] | 0.9 |  | 0.518 [0.073,3.69] | 0.5 |
| **Intermediate-risk factors (negative high-risk factors)** | | | | |  |  |  |
| **Any 1 of 4 factors:** |  |  |  |  |  |  |  |
| >2cm, LVSI, DSI≥2/3, non-SCC | 643 | 192 | 8.287 [1.119,61.387] | 0.01 |  | 6.421 [0.818,50.378] | 0.05 |
| >4cm, LVSI, DSI≥2/3, non-SCC | 778 | 208 | 1.976 [0.746,5.23] | 0.2 |  | 1.917 [0.636,5.78] | 0.2 |
| **Any 2 of 4 factors:** |  |  |  |  |  |  |  |
| >2cm, LVSI, DSI≥2/3, non-SCC | 623 | 181 | 0.771 [0.4,1.485] | 0.4 |  | 0.614 [0.294,1.283] | 0.2 |
| >4cm, LVSI, DSI≥2/3, non-SCC | 497 | 137 | 0.912 [0.452,1.84] | 0.8 |  | 0.726 [0.315,1.675] | 0.5 |
| **Any 3 of 4 factors:** |  |  |  |  |  |  |  |
| >2cm, LVSI, DSI≥2/3, non-SCC | 387 | 72 | 2.131 [0.72,6.305] | 0.2 |  | 3.216 [0.701,14.747] | 0.1 |
| >4cm, LVSI, DSI≥2/3, non-SCC | 212 | 36 | 1.857 [0.53,6.506] | 0.3 |  | 1.494 [0.406,5.507] | 0.5 |
| **All 4 factors** |  |  |  |  |  |  |  |
| >2cm, LVSI, DSI≥2/3, non-SCC | 63 | 14 | 0.918 [0.162,5.208] | 0.9 |  | 0.431 [0.058,3.232] | 0.4 |
| >4cm, LVSI, DSI≥2/3, non-SCC | 22 | 4 | 0.73 [0.066,8.065] | 0.8 |  | 0.289 [0.018,4.649] | 0.4 |

**Table S4**

Characteristics of stage ⅠA1(LVSI)-ⅡA1 CC patients, with or without adjuvant therapy (cohort 3).

| Characteristics | Cohort 3 (n=3986) | | |
| --- | --- | --- | --- |
|  | No-adjuvant treatment  (n=1814) | Adjuvant-treatment  (n=2172) | P |
| **Age** |  |  | 0.307 |
| Mean ± SD | 47±9.5 | 48.2±9.6 |  |
| **FIGO stage (%)** |  |  | <0.001 |
| 1A1 (LVSI) | 42(2.3%) | 17(0.8%) |  |
| 1A2 | 95(5.2%) | 17(0.8%) |  |
| 1B1 | 1402(77.3%) | 1401(64.5%) |  |
| 1B2 | 117(6.4%) | 371(17.1%) |  |
| 2A1 | 158(8.7%) | 366(16.9%) |  |
| **Comorbidity (%)** |  |  | 0.229 |
| No | 1550(85.4%) | 1826(84.1%) |  |
| Yes | 264(14.6%) | 346(15.9%) |  |
| **Surgery approach (%)** |  |  | 0.002 |
| MIS | 1404(77.4%) | 1769(81.4%) |  |
| OPEN | 410(22.6%) | 403(18.6%) |  |
| **Tumor size, cm (%)** |  |  | <0.001 |
| ≤2 | 1077(59.4%) | 463(21.3%) |  |
| (2,4] | 593(32.7%) | 983(45.3%) |  |
| >4 | 144(7.9%) | 726(33.4%) |  |
| **Histology (%)** |  |  | <0.001 |
| SCC | 1331(73.4%) | 1734(79.8%) |  |
| AC | 223(12.3%) | 254(11.7%) |  |
| AS | 87(4.8%) | 142(6.5%) |  |
| Rare type | 12(0.7%) | 26(1.2%) |  |
| Unknown | 161(8.9%) | 16(0.7%) |  |
| **DSI (%)** |  |  | <0.001 |
| Negative | 776(42.8%) | 257(11.8%) |  |
| <2/3 | 768(42.3%) | 505(23.3%) |  |
| ≥2/3 | 270(14.9%) | 1410(64.9%) |  |
| **LVSI (%)** |  |  | <0.001 |
| No | 1525(84.1%) | 835(38.4%) |  |
| Yes | 289(15.9%) | 1337(61.6%) |  |
| **Surgical margin (%)** |  |  | <0.001 |
| No | 1750(96.5%) | 1960(90.2%) |  |
| Yes | 64(3.5%) | 212(9.8%) |  |
| **parametrial invasion (%)** |  |  | <0.001 |
| No | 1781(98.2%) | 2007(92.4%) |  |
| Yes | 33(1.8%) | 165(7.6%) |  |
| **LN metastasis (%)** |  |  | <0.001 |
| No | 1738(95.8%) | 1568(72.2%) |  |
| Yes | 76(4.2%) | 604(27.8%) |  |
| **Metastasis site (%)** |  |  | <0.001 |
| No | 1738(95.8%) | 1568(72.2%) |  |
| Pelvic LN | 55(3%) | 460(21.2%) |  |
| Common iliac LN | 20(1.1%) | 117(5.4%) |  |
| Para-aortic LN | 1(0.1%) | 27(1.2%) |  |

**Table S5**

Characteristics of stage ⅠA1(LVSI)-ⅡA1 CC patients with or without adjuvant therapy (cohort 3), after propensity-score matching

| Characteristics | Adjuvant-treatment,  after matching (n=2015) | | |  | No-adjuvant-treatment,  after matching(n=3252) | | |
| --- | --- | --- | --- | --- | --- | --- | --- |
|  | MIS  (n=1612) | OPEN  (n=403) | P |  | MIS  (n=2439) | OPEN  (n=813) | P |
| **Age** |  |  |  |  |  |  | 0.098 |
| Mean ± SD | 48±9.7 | 48.3±9.8 | 0.635 |  | 47.1±9.5 | 47.6±9 |  |
| **FIGO stage (%)** |  |  | 0.055 |  |  |  | 0.024 |
| 1A1 (LVSI) | 7(0.4%) | 5(1.2%) |  |  | 18(1.5%) | 4(1%) |  |
| 1A2 | 15(0.9%) | 2(0.5%) |  |  | 40(3.3%) | 19(4.6%) |  |
| 1B1 | 1019(63.2%) | 242(60%) |  |  | 986(80.2%) | 301(73.4%) |  |
| 1B2 | 294(18.2%) | 66(16.4%) |  |  | 82(6.7%) | 33(8%) |  |
| 2A1 | 277(17.2%) | 88(21.8%) |  |  | 104(8.5%) | 53(12.9%) |  |
| **Comorbidity (%)** |  |  | 0.561 |  |  |  | 0.688 |
| No | 1363(84.6%) | 336(83.4%) |  |  | 1046(85%) | 352(85.9%) |  |
| Yes | 259(15.4%) | 67(16.6%) |  |  | 184(15%) | 58(14.1%) |  |
| **Tumor size, cm (%)** |  |  | 0.368 |  |  |  | 0.011 |
| ≤2 | 362(22.5%) | 100(24.8%) |  |  | 707(57.5%) | 205(50%) |  |
| (2,4] | 732(45.4%) | 187(46.4%) |  |  | 427(34.7%) | 158(38.5%) |  |
| >4 | 518(32.1%) | 116(28.8%) |  |  | 96(7.8%) | 47(11.5%) |  |
| **Histology (%)** |  |  | 0.088 |  |  |  | 0.558 |
| SCC | 1272(78.9%) | 319(79.2%) |  |  | 946(76.9%) | 321(78.3%) |  |
| AC | 193(12%) | 52(12.9%) |  |  | 169(13.7%) | 46(11.2%) |  |
| AS | 116(7.2%) | 21(5.2%) |  |  | 54(4.4%) | 22(5.4%) |  |
| Rare type | 22(1.4%) | 4(1%) |  |  | 7(0.6%) | 4(1%) |  |
| Unknown | 9(0.6%) | 7(1.7%) |  |  | 54(4.4%) | 17(4.1%) |  |
| **DSI (%)** |  |  | 0.947 |  |  |  | <0.001 |
| Negative | 196(12.2%) | 47(11.7%) |  |  | 474(38.5%) | 134(32.7%) |  |
| <2/3 | 375(23.3%) | 96(23.8%) |  |  | 590(48%) | 172(42%) |  |
| ≥2/3 | 1041(64.6%) | 260(64.5%) |  |  | 166(13.5%) | 104(25.4%) |  |
| **LVSI (%)** |  |  | 0.946 |  |  |  | 0.464 |
| No | 655(40.6%) | 163(40.4%) |  |  | 1033(84%) | 338(82.4%) |  |
| Yes | 957(59.4%) | 240(59.6%) |  |  | 197(16%) | 72(17.6%) |  |
| **Surgical margin (%)** |  |  | 0.821 |  |  |  | 0.809 |
| No | 1458(90.4%) | 363(90.1%) |  |  | 1191(96.8%) | 396(96.6%) |  |
| Yes | 154(9.6%) | 40(9.9%) |  |  | 39(3.2%) | 14(3.4%) |  |
| **Parametrial invasion (%)** |  |  | 0.191 |  |  |  | 0.477 |
| No | 1488(92.3%) | 364(90.3%) |  |  | 1207(98.1%) | 400(97.6%) |  |
| Yes | 124(7.7%) | 39(9.7%) |  |  | 23(1.9%) | 10(2.4%) |  |
| **LN metastasis (%)** |  |  | 0.001 |  |  |  | 0.619 |
| No | 1155(71.7%) | 256(63.5%) |  |  | 1180(95.9%) | 391(95.4%) |  |
| Yes | 457(28.3%) | 147(36.5%) |  |  | 50(4.1%) | 19(4.6%) |  |
| **Metastasis site (%)** |  |  | 0.017 |  |  |  | 0.434 |
| No | 1155(71.7%) | 256(63.5%) |  |  | 1180(95.9%) | 391(95.4%) |  |
| Pelvic LN | 347(21.5%) | 113(28%) |  |  | 33(2.7%) | 16(3.9%) |  |
| Common iliac LN | 89(5.5%) | 28(6.9%) |  |  | 16(1.3%) | 3(0.7%) |  |
| Para-aortic LN | 21(1.3%) | 6(6.9%) |  |  | 1(0.1%) | 0(0%) |  |

**Table S6**

Survival outcome comparisons between skilled and unskilled group, after propensity-score matching.

| Characteristics | MIS+OPEN (n=3027) | | |  | MIS (n=2755) | | | OPEN (n=272) | | |
| --- | --- | --- | --- | --- | --- | --- | --- | --- | --- | --- |
|  | Skilled  (n=2613) | Unskilled  (n=414) | P |  | Skilled  (n=2388) | Unskilled  (n=367) | P | Skilled  (n=225) | Unskilled  (n=47) | P |
| **Age** |  |  | 0.475 |  |  |  | 0.288 |  |  | 0.463 |
| Mean ± SD | 47.5±9.6 | 47.4±9.2 |  |  | 47.5±9.6 | 47.6±9.1 |  | 47.4±9.6 | 46.3±10.5 |  |
| **FIGO stage (%)** |  |  | 0.056 |  |  |  | 0.3 |  |  | 0.082 |
| 1A1 (LVSI) | 0(0) | 1(0.2%) |  |  | 0(0) | 0(0) |  | 0(0) | 1(2.1%) |  |
| 1A2 | 59(2.3%) | 6(1.4%) |  |  | 50(2.1%) | 3(0.8%) |  | 9(4%) | 3(6.4%) |  |
| 1B1 | 2354(90.1%) | 368(88.9%) |  |  | 2188(91.6%) | 340(92.6%) |  | 166(73.8%) | 28(59.6%) |  |
| 1B2 | 152(5.8%) | 28(6.8%) |  |  | 119(5%) | 17(4.6%) |  | 33(14.7%) | 11(23.4%) |  |
| 2A1 | 48(1.8%) | 11(2.7) |  |  | 31(1.3%) | 7(1.9%) |  | 17(7.6%) | 4(8.5%) |  |
| **Adjuvant therapy (%)** |  |  | 0.493 |  |  |  | 0.299 |  |  | 0.216 |
| No | 1069(40.9%) | 162(39.1%) |  |  | 999(41.8%) | 143(39%) |  | 70(31.3%) | 19(40.4%) |  |
| Yes | 1544(59.1%) | 252(60.9%) |  |  | 1389(58.2%) | 224(61%) |  | 155(68.9%) | 28(59.6%) |  |
| **Tumor size, cm (%)** |  |  | 0.615 |  |  |  | 0.544 |  |  | 0.918 |
| ≤2 | 992(38%) | 160(38.6%) |  |  | 918(38.4%) | 146(39.8%) |  | 74(32.9%) | 14(29.8%) |  |
| (2,4] | 1008(38.6%) | 150(36.2%) |  |  | 916(38.4%) | 130(35.4%) |  | 92(40.9%) | 20(42.6%) |  |
| >4 | 613(23.5%) | 104(25.1%) |  |  | 554(23.2%) | 91(24.8%) |  | 59(26.2%) | 13(27.7%) |  |
| **Histology (%)** |  |  | 0.984 |  |  |  | 0.743 |  |  | 0.125 |
| SCC | 1971(75.4%) | 314(75.8%) |  |  | 1798(75.3%) | 281(76.6%) |  | 173(76.9%) | 33(70.2%) |  |
| AC | 313(12%) | 46(11.1%) |  |  | 297(12.4%) | 37(10.1%) |  | 16(7.1%) | 9(19.1%) |  |
| AS | 156(6%) | 25(6%) |  |  | 142(5.9%) | 23(6.3%) |  | 14(6.2%) | 2(4.3%) |  |
| Rare type | 21(0.8%) | 4(1%) |  |  | 20(0.8%) | 4(1.1%) |  | 1(0.4%) | 0(0) |  |
| Unknown | 152(5.8%) | 25(6%) |  |  | 131(5.5%) | 22(6%) |  | 21(9.3%) | 3(6.4%) |  |
| **DSI (%)** |  |  | 0.546 |  |  |  | 0.191 |  |  | 0.103 |
| Negative | 660(25.3%) | 115(27.8%) |  |  | 607(25.4%) | 109(29.7%) |  | 53(23.6%) | 6(12.8%) |  |
| <2/3 | 714(27.3%) | 108(26.1%) |  |  | 714(29.9%) | 108(29.4%) |  | 172(76.4%) | 41(87.2%) |  |
| ≥2/3 | 1239(47.4%) | 191(46.1%) |  |  | 1067(44.7%) | 150(40.9%) |  | 0(0) | 0(0) |  |
| **LVSI (%)** |  |  | 0.239 |  |  |  | 0.107 |  |  | 0.355 |
| No | 1532(58.6%) | 230(55.6%) |  |  | 1395(58.4%) | 198(54%) |  | 137(60.9%) | 32(68.1%) |  |
| Yes | 1081(41.4%) | 184(44.4%) |  |  | 993(41.6%) | 169(46%) |  | 88(39.1%) | 15(31.9%) |  |
| **Surgical margin (%)** |  |  | 0.292 |  |  |  | 0.144 |  |  | 0.328 |
| No | 2424(92.8%) | 378(91.3%) |  |  | 2218(92.9%) | 333(90.7%) |  | 206(91.6%) | 45(95.7%) |  |
| Yes | 189(7.2%) | 36(8.7%) |  |  | 170(7.1%) | 34(9.3%) |  | 19(8.4%) | 2(4.3%) |  |
| **Parametrial invasion (%)** |  |  | 0.524 |  |  |  | 0.779 |  |  | 0.263 |
| No | 2487(95.2%) | 397(95.9%) |  |  | 2276(95.3%) | 351(95.6%) |  | 211(93.8%) | 46(97.9%) |  |
| Yes | 126(4.8%) | 17(4.1%) |  |  | 112(4.7%) | 16(4.4%) |  | 14(6.2%) | 1(2.1%) |  |
| **LN metastasis (%)** |  |  | 0.151 |  |  |  | 0.078 |  |  | 0.485 |
| No | 2206(84.4%) | 338(81.6%) |  |  | 2019(84.5%) | 297(80.9%) |  | 187(83.1%) | 41(87.2%) |  |
| Yes | 407(15.6%) | 76(18.4%) |  |  | 369(15.5%) | 70(19.1%) |  | 38(16.9%) | 6(12.8%) |  |
